# Supplementary figures and images for: Effects of preservation method on canine (Canis lupus familiaris) fecal microbiota
Source: PeerJ. 2018 May 23;6:e4827. doi: 10.7717/peerj.4827 (PMC5970549; doi:10.7717/peerj.4827)

**25°C**

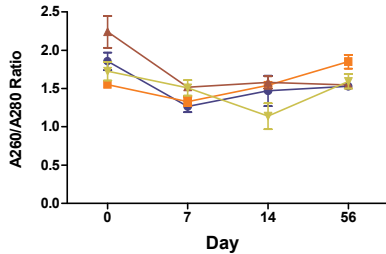

**4°C**

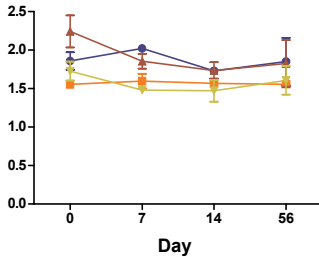

**-80°C**

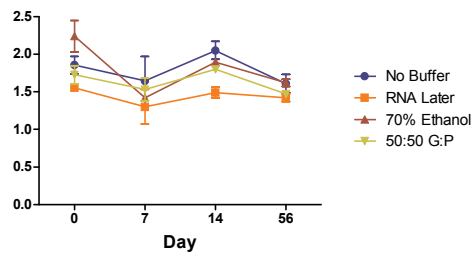

Supplement: Figure S1 — Across all buffers and temperatures, DNA purity ±standard error did not change significantly over time. [file peerj-06-4827-s001.pdf]

25°C

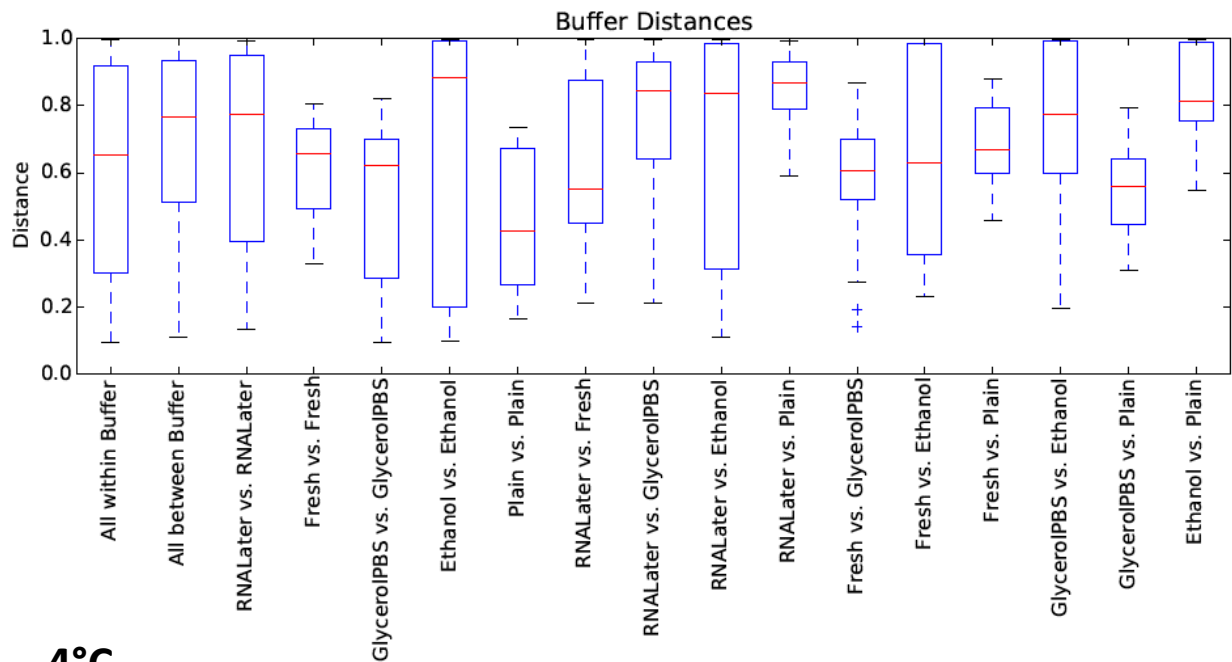

4°C

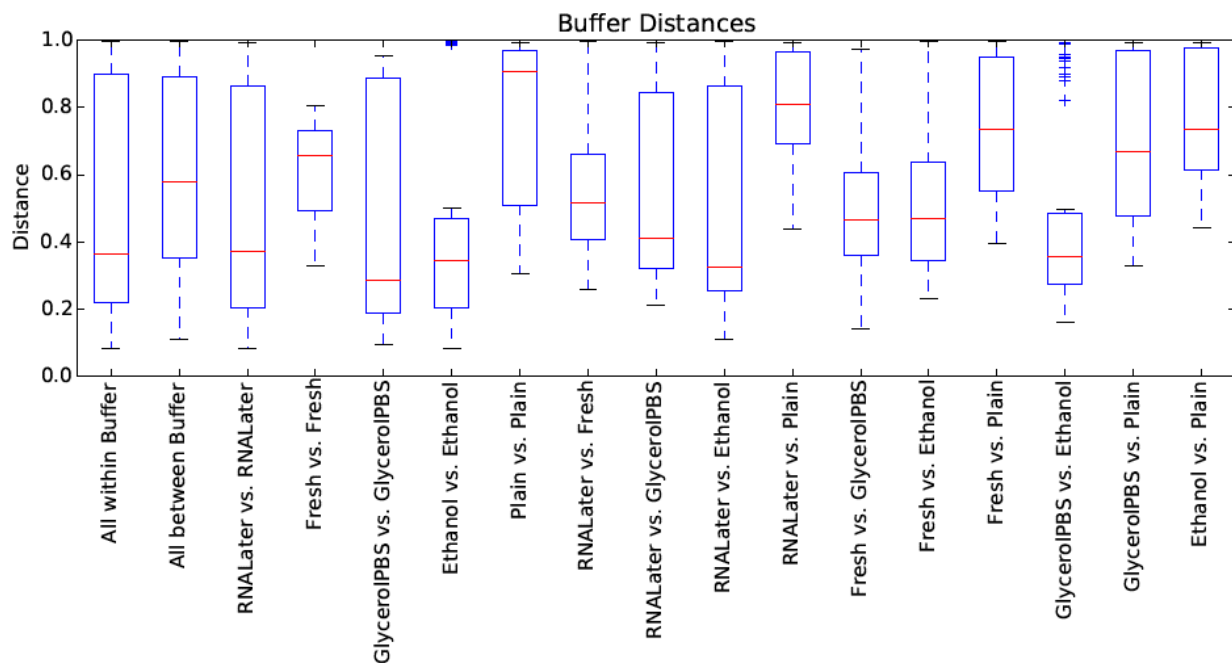

-80°C

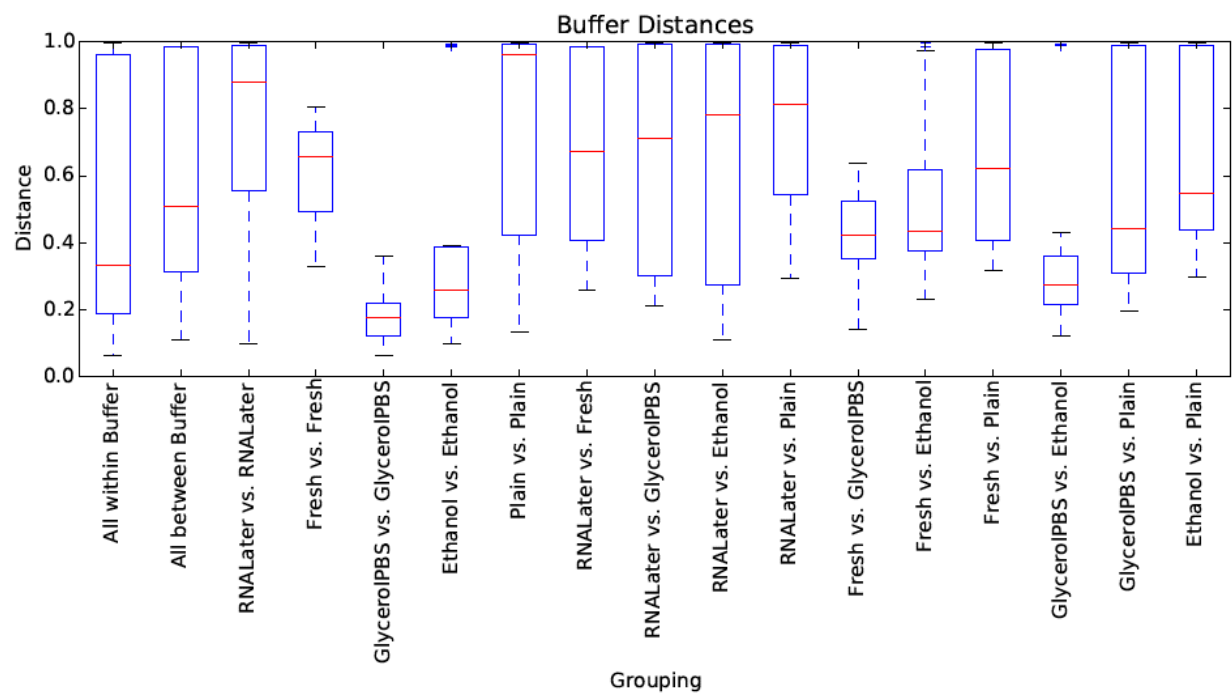

Supplement: Figure S5 — Boxplot of Bray Curtis dissimilarities between sample groups representing median, lower quartile, and upper quartile distances bound between 0 and 1. [file peerj-06-4827-s005.pdf]

# Qubit vs. Nanodrop

25°C

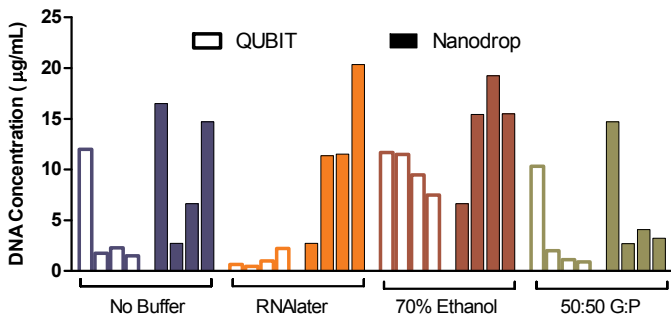

4°C

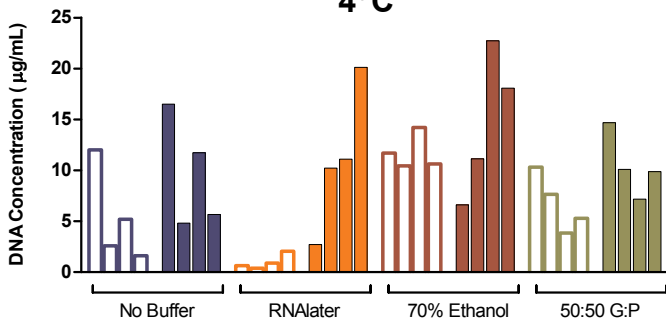

-80°C

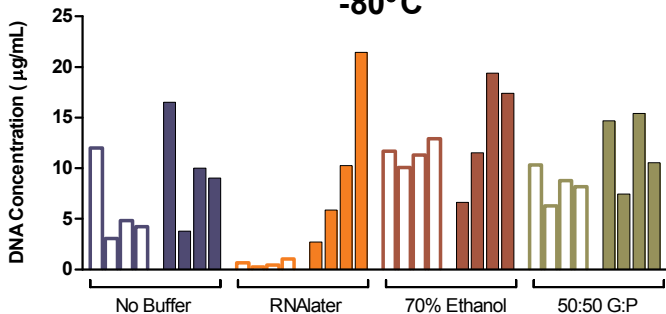

Supplement: Figure S6 — Across all buffers and temperatures, DNA detection by Nanodrop yielded much higher concentrations than that of QUBIT, particularly in that of RNA later samples. [file peerj-06-4827-s006.pdf]
