# Supplementary material for: Effects of preservation method on canine (Canis lupus familiaris) fecal microbiota
Source: PeerJ. 2018 May 23;6:e4827. doi: 10.7717/peerj.4827 (PMC5970549; doi:10.7717/peerj.4827)
Supplement: Figure S2 — Significant effects were found according to storage buffer ( F-value = 3.07, DF = 3, P = 0.03). *** p < 0.001, ** p < 0.01, * p < 0.05. [file peerj-06-4827-s002.pdf]

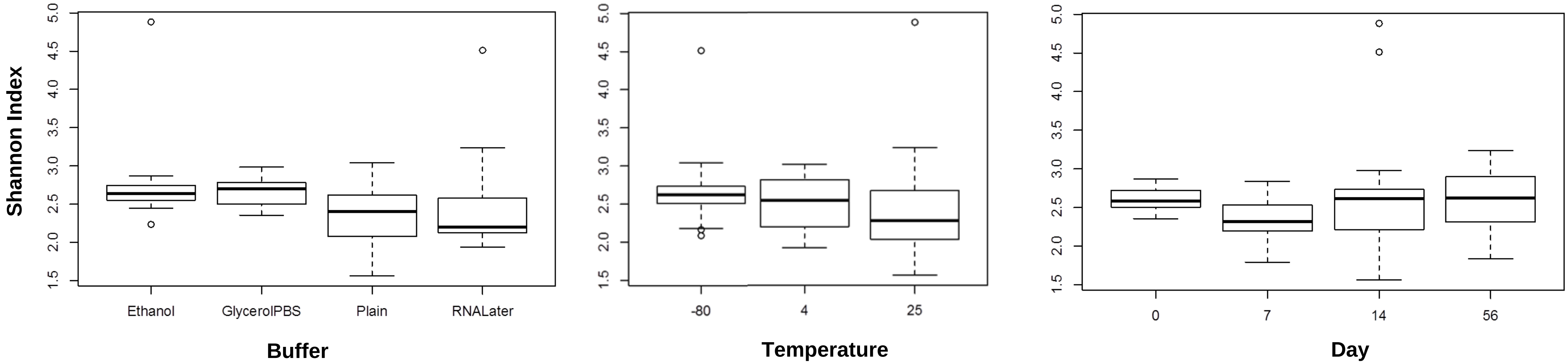

Variables	Df	Sum Sq	Mean Sq	F value	Pr(>F)
Buffer	3	1.884	0.6281	3.073	0.0339 *
Temperature	1	0.451	0.451	2.206	0.1424
Day	1	0.142	0.142	0.693	0.4081
Buffer x Temperature	3	1.153	0.3843	1.880	0.1419
Buffer x Day	3	0.572	0.1907	0.933	0.4302
Temperature x Day	1	0.116	0.1162	0.568	0.4536
Buffer x Temperature x Day	3	0.612	0.2041	0.998	0.3995
