# Supplementary material for: Effects of preservation method on canine (Canis lupus familiaris) fecal microbiota
Source: PeerJ. 2018 May 23;6:e4827. doi: 10.7717/peerj.4827 (PMC5970549; doi:10.7717/peerj.4827)
Supplement: Figure S3 — Significant effects were found according to storage buffer (F-value = 12.4, DF = 3, P < 0.00001), duration of sample storage (F-value = 10.8, DF = 1, P = 0.0016), the interaction between storage buffer and temperature (F-value = 3.443, DF = 3, P = 0.22), and the interaction between storage buffer and duration of sample storage (F-value = 9.67, DF = 3, P < 0.00001). *** p < 0.001, **p¡0.01, *p < 0.05. [file peerj-06-4827-s003.pdf]

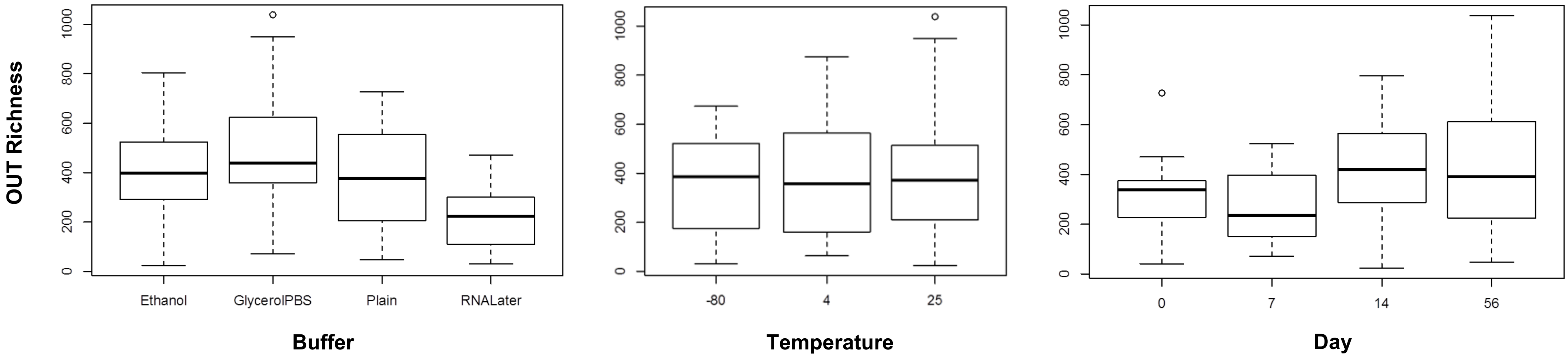

Variables	Df	Sum Sq	Mean Sq	F value	Pr(>F)
Buffer	3	921397	307132	12.421	0.000001 ***
Temperature	1	20793	20793	0.841	0.362580
Day	1	268202	268202	10.846	0.001620 **
Buffer x Temperature	3	255404	85135	3.443	0.021800 *
Buffer x Day	3	716960	238987	9.665	0.000024 ***
Temperature x Day	1	54120	54120	2.189	0.143940
Buffer x Temperature x Day	3	183684	61228	2.476	0.069330
