# Supplementary material for: Effects of preservation method on canine (Canis lupus familiaris) fecal microbiota
Source: PeerJ. 2018 May 23;6:e4827. doi: 10.7717/peerj.4827 (PMC5970549; doi:10.7717/peerj.4827)
Supplement: Figure S4 — There were significant effects associated with interactions between storage buffer and storage temperature (F-value = 3.98, DF = 3, P = 0.01), storage buffer and duration of sample storage (F-value = 4.9, DF = 3, P = 0.004), and buffer, storage temperature, and duration of sample storage (F-value = 3.1, DF = 3, P = 0.03). *** p < 0.001, ** p < 0.01, * p < 0.05. [file peerj-06-4827-s004.pdf]

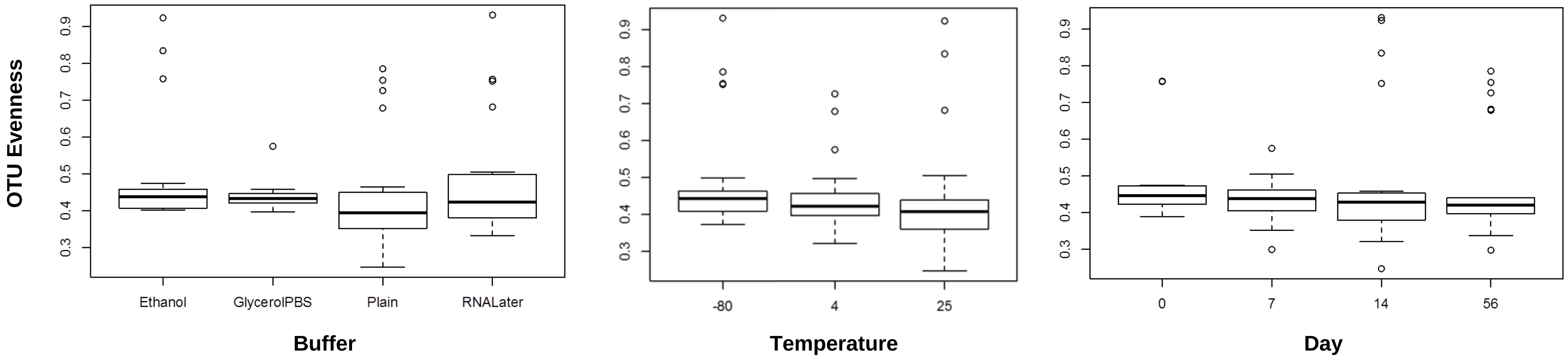

Variables	Df	Sum Sq	Mean Sq	F value	Pr(>F)
Buffer	3	0.0440	0.01466	1.027	0.38654
Temperature	1	0.0327	0.03273	2.294	0.13481
Day	1	0.0003	0.00029	0.020	0.88667
Buffer x Temperature	3	0.1702	0.05674	3.976	0.01161 *
Buffer x Day	3	0.2091	0.06970	4.884	0.00404 **
Temperature x Day	1	0.0014	0.00135	0.095	0.75933
Buffer x Temperature x Day	3	0.1314	0.04381	3.070	0.03402 *
