# Supplementary material for: Effects of preservation method on canine (Canis lupus familiaris) fecal microbiota
Source: PeerJ. 2018 May 23;6:e4827. doi: 10.7717/peerj.4827 (PMC5970549; doi:10.7717/peerj.4827)
Supplement: Table S1 — The difference between the means (diff), upper and lower levels of the 95% confidence interval around that mean difference, and p-values adjusted using Tukey were determined on R software. Significant interactions (bold) were considered when p < 0.05. [file peerj-06-4827-s007.docx]

| **Buffer** | **diff** | **lwr** | **upr** | **p adj** |
| --- | --- | --- | --- | --- |
| GlycerolPBS-Ethanol | 117.52 | 16.75 | 218.29 | **0.0167** |
| Plain-Ethanol | -49.14 | -144.46 | 46.18 | 0.5178 |
| RNALater-Ethanol | -188.76 | -284.08 | -93.44 | **<0.0001** |
| Plain-GlycerolPBS | -166.66 | -267.44 | -65.89 | **0.0004** |
| RNALater-GlycerolPBS | -306.28 | -407.06 | -205.51 | **<0.0001** |
| RNALater-Plain | -139.62 | -234.94 | -44.30 | **0.0018** |
