# Supplementary material for: Effects of preservation method on canine (Canis lupus familiaris) fecal microbiota
Source: PeerJ. 2018 May 23;6:e4827. doi: 10.7717/peerj.4827 (PMC5970549; doi:10.7717/peerj.4827)
Supplement: Table S2 — The difference between the means (diff), upper and lower levels of the 95% confidence interval around that mean difference, and p-values adjusted using Tukey were determined on R software. Significant interactions (bold) were considered when p < 0.05. [file peerj-06-4827-s008.docx]

| **Buffer** | **diff** | **lwr** | **upr** | **p adj** |
| --- | --- | --- | --- | --- |
| GlycerolPBS-Ethanol | 117.52 | 16.75 | 218.29 | **0.0167** |
| Plain-Ethanol | -49.14 | -144.46 | 46.18 | 0.5178 |
| RNALater-Ethanol | -188.76 | -284.08 | -93.44 | **<0.0001** |
| Plain-GlycerolPBS | -166.66 | -267.44 | -65.89 | **0.0004** |
| RNALater-GlycerolPBS | -306.28 | -407.06 | -205.51 | **<0.0001** |
| RNALater-Plain | -139.62 | -234.94 | -44.30 | **0.0018** |

| **Days of storage** | **diff** | **lwr** | **upr** | **p adj** |
| --- | --- | --- | --- | --- |
| 7-0 | -103.62 | -214.47 | 7.22 | 0.0743 |
| 14-0 | 29.99 | -79.22 | 139.19 | 0.8820 |
| 56-0 | 70.91 | -39.93 | 181.76 | 0.3296 |
| 14-7 | 133.61 | 42.44 | 224.78 | **0.0018** |
| 56-7 | 174.54 | 81.41 | 267.66 | **0.0001** |
| 56-14 | 40.93 | -50.24 | 132.09 | 0.6284 |

| **Buffer x Temperature** | **diff** | **lwr** | **upr** | **p adj** |
| --- | --- | --- | --- | --- |
| RNALater:-80-Ethanol:-80 | -365.67 | -608.55 | -122.79 | **0.0002** |
| Ethanol:1-Ethanol:-80 | -318.33 | -615.80 | -20.86 | **0.0259** |
| RNALater:4-Ethanol:-80 | -258.16 | -501.04 | -15.28 | **0.0277** |
| RNALater:25-Ethanol:-80 | -308.23 | -551.11 | -65.35 | **0.0034** |
| RNALater:-80-GlycerolPBS:-80 | -316.97 | -571.71 | -62.24 | **0.0044** |
| RNALater:25-GlycerolPBS:-80 | -259.54 | -514.27 | -4.80 | **0.0421** |
| GlycerolPBS:25-Plain:-80 | 419.57 | 164.83 | 674.30 | **<0.0001** |
| Ethanol:4-RNALater:-80 | 374.34 | 131.46 | 617.22 | **0.0002** |
| GlycerolPBS:4-RNALater:-80 | 332.80 | 61.25 | 604.34 | **0.0053** |
| GlycerolPBS:25-RNALater:-80 | 560.40 | 305.67 | 815.14 | **<0.0001** |
| Plain:25-RNALater:-80 | 301.77 | 58.89 | 544.65 | **0.0045** |
| Ethanol:4-Ethanol:1 | 327.00 | 29.53 | 624.47 | **0.0195** |
| GlycerolPBS:25-Ethanol:1 | 513.06 | 205.84 | 820.29 | **<0.0001** |
| GlycerolPBS:25-GlycerolPBS:1 | 342.34 | 35.11 | 649.56 | **0.0168** |
| GlycerolPBS:25-RNALater:1 | 410.40 | 103.17 | 717.62 | **0.0016** |
| RNALater:4-Ethanol:4 | -266.83 | -509.71 | -23.95 | **0.0196** |
| RNALater:25-Ethanol:4 | -316.90 | -559.78 | -74.02 | **0.0023** |
| RNALater:25-GlycerolPBS:4 | -275.36 | -546.91 | -3.81 | **0.0440** |
| GlycerolPBS:25-Plain:4 | 409.56 | 154.83 | 664.30 | **0.0001** |
| GlycerolPBS:25-Ethanol:25 | 423.46 | 168.73 | 678.20 | **<0.0001** |
| Plain:25-GlycerolPBS:25 | -258.63 | -513.37 | -3.90 | **0.0435** |
| RNALater:25-GlycerolPBS:25 | -502.96 | -757.70 | -248.23 | **<0.0001** |
| RNALater:25-Plain:25 | -244.33 | -487.21 | -1.45 | **0.0473** |

| **Buffer x Days of storage** | **diff** | **lwr** | **upr** | **p adj** |
| --- | --- | --- | --- | --- |
| RNALater:7-GlycerolPBS:0 | -391.03 | -688.49 | -93.56 | **0.0020** |
| GlycerolPBS:56-Plain:0 | 431.03 | 109.73 | 752.34 | **0.0015** |
| GlycerolPBS:14-RNALater:0 | 315.22 | 17.75 | 612.69 | **0.0286** |
| Plain:14-RNALater:0 | 331.44 | 33.97 | 628.90 | **0.0168** |
| Ethanol:56-RNALater:0 | 321.99 | 24.52 | 619.46 | **0.0230** |
| GlycerolPBS:56-RNALater:0 | 570.65 | 249.35 | 891.95 | **<0.0001** |
| RNALater:7-Ethanol:7 | -244.10 | -486.98 | -1.21 | **0.0478** |
| GlycerolPBS:56-Ethanol:7 | 415.03 | 143.48 | 686.58 | **0.0002** |
| GlycerolPBS:14-GlycerolPBS:7 | 287.88 | 16.33 | 559.43 | **0.0285** |
| Plain:14-GlycerolPBS:7 | 304.09 | 32.55 | 575.64 | **0.0159** |
| Ethanol:56-GlycerolPBS:7 | 294.65 | 23.10 | 566.20 | **0.0224** |
| GlycerolPBS:56-GlycerolPBS:7 | 543.31 | 245.85 | 840.78 | **<0.0001** |
| GlycerolPBS:14-Plain:7 | 251.40 | 8.52 | 494.29 | **0.0361** |
| Plain:14-Plain:7 | 267.62 | 24.74 | 510.50 | **0.0190** |
| Ethanol:56-Plain:7 | 258.17 | 15.29 | 501.05 | **0.0277** |
| GlycerolPBS:56-Plain:7 | 506.84 | 235.29 | 778.39 | **<0.0001** |
| GlycerolPBS:14-RNALater:7 | 403.69 | 160.81 | 646.57 | **<0.0001** |
| Plain:14-RNALater:7 | 419.90 | 177.02 | 662.79 | **<0.0001** |
| Ethanol:56-RNALater:7 | 410.46 | 167.58 | 653.34 | **<0.0001** |
| GlycerolPBS:56-RNALater:7 | 659.12 | 387.57 | 930.67 | **<0.0001** |
| Plain:14-Ethanol:14 | 246.02 | 3.14 | 488.90 | **0.0444** |
| GlycerolPBS:56-Ethanol:14 | 485.24 | 213.69 | 756.79 | **<0.0001** |
| RNALater:14-GlycerolPBS:14 | -296.40 | -539.29 | -53.52 | **0.0056** |
| Plain:56-GlycerolPBS:14 | -294.87 | -537.76 | -51.99 | **0.0060** |
| RNALater:56-GlycerolPBS:14 | -248.83 | -491.71 | -5.95 | **0.0399** |
| RNALater:14-Plain:14 | -312.62 | -555.50 | -69.74 | **0.0028** |
| Plain:56-Plain:14 | -311.09 | -553.97 | -68.21 | **0.0030** |
| RNALater:56-Plain:14 | -265.04 | -507.92 | -22.16 | **0.0211** |
| Ethanol:56-RNALater:14 | 303.17 | 60.29 | 546.05 | **0.0042** |
| GlycerolPBS:56-RNALater:14 | 551.84 | 280.29 | 823.39 | **<0.0001** |
| Plain:56-Ethanol:56 | -301.64 | -544.52 | -58.76 | **0.0045** |
| RNALater:56-Ethanol:56 | -255.60 | -498.48 | -12.71 | **0.0307** |
| Plain:56-GlycerolPBS:56 | -550.31 | -821.86 | -278.76 | **<0.0001** |
| RNALater:56-GlycerolPBS:56 | -504.26 | -775.81 | -232.71 | **<0.0001** |
