# Supplementary material for: Effects of preservation method on canine (Canis lupus familiaris) fecal microbiota
Source: PeerJ. 2018 May 23;6:e4827. doi: 10.7717/peerj.4827 (PMC5970549; doi:10.7717/peerj.4827)
Supplement: Table S3 — The difference between the means (diff), upper and lower levels of the 95% confidence interval around that mean difference, and p-values adjusted using Tukey were determined on R software. Significant interactions (bold) were considered when p < 0.05. [file peerj-06-4827-s009.docx]

| **Buffer x Temperature** | **diff** | **lwr** | **upr** | **p adj** |
| --- | --- | --- | --- | --- |
| Plain:25-Plain:-80 | -0.2214 | -0.3779 | -0.0649 | **0.0007** |
| Plain:25-RNALater:-80 | -0.2468 | -0.4033 | -0.0903 | **0.0001** |
| Plain:25-Ethanol:1 | -0.2483 | -0.4400 | -0.0566 | **0.0025** |
| Plain:25-RNALater:1 | -0.2286 | -0.4203 | -0.0369 | **0.0076** |
| Plain:25-Plain:4 | -0.1663 | -0.3228 | -0.0098 | **0.0278** |
| Plain:25-Ethanol:25 | -0.2519 | -0.4084 | -0.0953 | **0.0001** |

| **Buffer x Days of storage** | **diff** | **lwr** | **upr** | **p adj** |
| --- | --- | --- | --- | --- |
| Ethanol:14-Plain:7 | 0.2145 | 0.0580 | 0.3710 | **0.0011** |
| Plain:56-Plain:7 | 0.2094 | 0.0529 | 0.3659 | **0.0016** |
| GlycerolPBS:14-Ethanol:14 | -0.1621 | -0.3186 | -0.0056 | **0.0360** |
| Plain:14-Ethanol:14 | -0.2415 | -0.3980 | -0.0850 | **0.0002** |
| Ethanol:56-Ethanol:14 | -0.1673 | -0.3238 | -0.0108 | **0.0261** |
| GlycerolPBS:56-Ethanol:14 | -0.1858 | -0.3608 | -0.0108 | **0.0280** |
| Plain:56-GlycerolPBS:14 | 0.1569 | 0.0004 | 0.3135 | **0.0488** |
| RNALater:14-Plain:14 | 0.1788 | 0.0223 | 0.3353 | **0.0126** |
| Plain:56-Plain:14 | 0.2363 | 0.0798 | 0.3929 | **0.0002** |
| Plain:56-Ethanol:56 | 0.1622 | 0.0057 | 0.3187 | **0.0357** |
| Plain:56-GlycerolPBS:56 | 0.1807 | 0.0057 | 0.3557 | **0.0370** |

| **Buffer x Temp x Days of storage** | **diff** | **lwr** | **upr** | **p adj** |
| --- | --- | --- | --- | --- |
| Ethanol:25:14-Ethanol:1:0 | 0.3107 | 0.0085 | 0.6130 | **0.0363** |
| RNALater:-80:14-GlycerolPBS:1:0 | 0.4252 | 0.1229 | 0.7274 | **0.0003** |
| Ethanol:25:14-GlycerolPBS:1:0 | 0.4625 | 0.1603 | 0.7647 | **<0.0001** |
| Plain:-80:56-GlycerolPBS:1:0 | 0.3535 | 0.0513 | 0.6557 | **0.0064** |
| RNALater:-80:14-Plain:1:0 | 0.4139 | 0.1116 | 0.7161 | **0.0005** |
| Ethanol:25:14-Plain:1:0 | 0.4512 | 0.1490 | 0.7535 | **0.0001** |
| Plain:-80:56-Plain:1:0 | 0.3422 | 0.0400 | 0.6445 | **0.0103** |
| Ethanol:25:14-RNALater:1:0 | 0.3304 | 0.0282 | 0.6327 | **0.0167** |
| RNALater:-80:14-GlycerolPBS:-80:7 | 0.4120 | 0.0809 | 0.7430 | **0.0024** |
| Ethanol:25:14-GlycerolPBS:-80:7 | 0.4493 | 0.1183 | 0.7804 | **0.0005** |
| Plain:-80:56-GlycerolPBS:-80:7 | 0.3403 | 0.0093 | 0.6714 | **0.0363** |
| RNALater:-80:14-Plain:-80:7 | 0.3878 | 0.0567 | 0.7189 | **0.0063** |
| Ethanol:25:14-Plain:-80:7 | 0.4251 | 0.0941 | 0.7562 | **0.0014** |
| RNALater:-80:14-RNALater:-80:7 | 0.3612 | 0.0302 | 0.6923 | **0.0172** |
| Ethanol:25:14-RNALater:-80:7 | 0.3986 | 0.0675 | 0.7297 | **0.0041** |
| RNALater:-80:14-Ethanol:4:7 | 0.3900 | 0.0589 | 0.7211 | **0.0058** |
| Ethanol:25:14-Ethanol:4:7 | 0.4274 | 0.0963 | 0.7585 | **0.0013** |
| RNALater:-80:14-Plain:4:7 | 0.4527 | 0.1217 | 0.7838 | **0.0005** |
| Ethanol:25:14-Plain:4:7 | 0.4901 | 0.1590 | 0.8212 | **0.0001** |
| Plain:-80:56-Plain:4:7 | 0.3811 | 0.0500 | 0.7122 | **0.0081** |
| RNALater:-80:14-RNALater:4:7 | 0.3861 | 0.0550 | 0.7171 | **0.0067** |
| Ethanol:25:14-RNALater:4:7 | 0.4234 | 0.0924 | 0.7545 | **0.0015** |
| RNALater:-80:14-Ethanol:25:7 | 0.4363 | 0.1053 | 0.7674 | **0.0009** |
| Ethanol:25:14-Ethanol:25:7 | 0.4737 | 0.1426 | 0.8048 | **0.0002** |
| Plain:-80:56-Ethanol:25:7 | 0.3647 | 0.0336 | 0.6958 | **0.0151** |
| Ethanol:25:14-GlycerolPBS:25:7 | 0.4312 | 0.0257 | 0.8367 | **0.0240** |
| RNALater:-80:14-Plain:25:7 | 0.5157 | 0.1846 | 0.8468 | **<0.0001** |
| Ethanol:25:14-Plain:25:7 | 0.5531 | 0.2220 | 0.8841 | **<0.0001** |
| Plain:-80:56-Plain:25:7 | 0.4441 | 0.1130 | 0.7751 | **0.0007** |
| Plain:4:56-Plain:25:7 | 0.3766 | 0.0455 | 0.7076 | **0.0097** |
| RNALater:-80:14-RNALater:25:7 | 0.3763 | 0.0452 | 0.7074 | **0.0098** |
| Ethanol:25:14-RNALater:25:7 | 0.4137 | 0.0826 | 0.7448 | **0.0023** |
| RNALater:-80:14-Ethanol:-80:14 | 0.3886 | 0.0575 | 0.7196 | **0.0061** |
| Ethanol:25:14-Ethanol:-80:14 | 0.4259 | 0.0949 | 0.7570 | **0.0014** |
| RNALater:-80:14-GlycerolPBS:-80:14 | 0.4000 | 0.0689 | 0.7310 | **0.0039** |
| Ethanol:25:14-GlycerolPBS:-80:14 | 0.4373 | 0.1063 | 0.7684 | **0.0009** |
| RNALater:-80:14-Plain:-80:14 | 0.4402 | 0.1092 | 0.7713 | **0.0008** |
| Ethanol:25:14-Plain:-80:14 | 0.4776 | 0.1465 | 0.8087 | **0.0002** |
| Plain:-80:56-Plain:-80:14 | 0.3686 | 0.0375 | 0.6997 | **0.0130** |
| Ethanol:4:14-RNALater:-80:14 | -0.4187 | -0.7498 | -0.0877 | **0.0019** |
| GlycerolPBS:4:14-RNALater:-80:14 | -0.3886 | -0.7197 | -0.0575 | **0.0061** |
| Plain:4:14-RNALater:-80:14 | -0.4770 | -0.8081 | -0.1459 | **0.0002** |
| RNALater:4:14-RNALater:-80:14 | -0.4616 | -0.7927 | -0.1305 | **0.0003** |
| GlycerolPBS:25:14-RNALater:-80:14 | -0.4084 | -0.7394 | -0.0773 | **0.0028** |
| Plain:25:14-RNALater:-80:14 | -0.5294 | -0.8605 | -0.1984 | **<0.0001** |
| RNALater:25:14-RNALater:-80:14 | -0.4883 | -0.8194 | -0.1572 | **0.0001** |
| Ethanol:-80:56-RNALater:-80:14 | -0.4249 | -0.7559 | -0.0938 | **0.0014** |
| GlycerolPBS:-80:56-RNALater:-80:14 | -0.4441 | -0.8495 | -0.0386 | **0.0163** |
| RNALater:-80:56-RNALater:-80:14 | -0.4623 | -0.7934 | -0.1312 | **0.0003** |
| Ethanol:4:56-RNALater:-80:14 | -0.4296 | -0.7607 | -0.0985 | **0.0012** |
| RNALater:4:56-RNALater:-80:14 | -0.4314 | -0.7625 | -0.1004 | **0.0011** |
| Ethanol:25:56-RNALater:-80:14 | -0.4143 | -0.7454 | -0.0832 | **0.0022** |
| GlycerolPBS:25:56-RNALater:-80:14 | -0.4308 | -0.7619 | -0.0997 | **0.0011** |
| Plain:25:56-RNALater:-80:14 | -0.5237 | -0.8548 | -0.1926 | **<0.0001** |
| Ethanol:25:14-Ethanol:4:14 | 0.4561 | 0.1250 | 0.7872 | **0.0004** |
| Plain:-80:56-Ethanol:4:14 | 0.3471 | 0.0160 | 0.6782 | **0.0286** |
| Ethanol:25:14-GlycerolPBS:4:14 | 0.4260 | 0.0949 | 0.7571 | **0.0014** |
| Ethanol:25:14-Plain:4:14 | 0.5143 | 0.1833 | 0.8454 | **<0.0001** |
| Plain:-80:56-Plain:4:14 | 0.4053 | 0.0743 | 0.7364 | **0.0032** |
| Plain:4:56-Plain:4:14 | 0.3379 | 0.0068 | 0.6689 | **0.0396** |
| Ethanol:25:14-RNALater:4:14 | 0.4990 | 0.1679 | 0.8300 | **0.0001** |
| Plain:-80:56-RNALater:4:14 | 0.3900 | 0.0589 | 0.7210 | **0.0058** |
| GlycerolPBS:25:14-Ethanol:25:14 | -0.4457 | -0.7768 | -0.1146 | **0.0006** |
| Plain:25:14-Ethanol:25:14 | -0.5668 | -0.8979 | -0.2357 | **<0.0001** |
| RNALater:25:14-Ethanol:25:14 | -0.5256 | -0.8567 | -0.1946 | **<0.0001** |
| Ethanol:-80:56-Ethanol:25:14 | -0.4622 | -0.7933 | -0.1312 | **0.0003** |
| GlycerolPBS:-80:56-Ethanol:25:14 | -0.4814 | -0.8869 | -0.0759 | **0.0051** |
| RNALater:-80:56-Ethanol:25:14 | -0.4997 | -0.8307 | -0.1686 | **0.0001** |
| Ethanol:4:56-Ethanol:25:14 | -0.4670 | -0.7981 | -0.1359 | **0.0003** |
| GlycerolPBS:4:56-Ethanol:25:14 | -0.4386 | -0.8441 | -0.0331 | **0.0192** |
| RNALater:4:56-Ethanol:25:14 | -0.4688 | -0.7999 | -0.1377 | **0.0002** |
| Ethanol:25:56-Ethanol:25:14 | -0.4517 | -0.7828 | -0.1206 | **0.0005** |
| GlycerolPBS:25:56-Ethanol:25:14 | -0.4682 | -0.7993 | -0.1371 | **0.0002** |
| Plain:25:56-Ethanol:25:14 | -0.5611 | -0.8922 | -0.2300 | **<0.0001** |
| RNALater:25:56-Ethanol:25:14 | -0.3542 | -0.6853 | -0.0231 | **0.0222** |
| Plain:-80:56-GlycerolPBS:25:14 | 0.3367 | 0.0056 | 0.6678 | **0.0412** |
| Plain:-80:56-Plain:25:14 | 0.4578 | 0.1267 | 0.7889 | **0.0004** |
| Plain:4:56-Plain:25:14 | 0.3903 | 0.0592 | 0.7214 | **0.0057** |
| Plain:-80:56-RNALater:25:14 | 0.4166 | 0.0856 | 0.7477 | **0.0020** |
| Plain:4:56-RNALater:25:14 | 0.3492 | 0.0181 | 0.6802 | **0.0266** |
| Plain:-80:56-Ethanol:-80:56 | 0.3532 | 0.0222 | 0.6843 | **0.0230** |
| RNALater:-80:56-Plain:-80:56 | -0.3907 | -0.7217 | -0.0596 | **0.0056** |
| Ethanol:4:56-Plain:-80:56 | -0.3580 | -0.6891 | -0.0269 | **0.0193** |
| RNALater:4:56-Plain:-80:56 | -0.3598 | -0.6909 | -0.0287 | **0.0181** |
| Ethanol:25:56-Plain:-80:56 | -0.3427 | -0.6738 | -0.0116 | **0.0335** |
| GlycerolPBS:25:56-Plain:-80:56 | -0.3592 | -0.6903 | -0.0281 | **0.0185** |
| Plain:25:56-Plain:-80:56 | -0.4521 | -0.7832 | -0.1210 | **0.0005** |
| Plain:25:56-Plain:4:56 | -0.3846 | -0.7157 | -0.0535 | **0.0071** |
