# Supplementary material for: Effects of preservation method on canine (Canis lupus familiaris) fecal microbiota
Source: PeerJ. 2018 May 23;6:e4827. doi: 10.7717/peerj.4827 (PMC5970549; doi:10.7717/peerj.4827)
Supplement: Table S4 — Significance testing of Bray Curtis dissimilarity was performed using a two-sided Student’s two-sample t-test. T-statistics and P-values including Bonferonni correction between buffers were evaluated with sample storage at 25 °C, 4 °C, and −80 °C. [file peerj-06-4827-s010.docx]

| **Group 1** | **Group 2** | **t statistic** | **Parametric p-value** | **Parametric p-value (Bonferroni-corrected)** | **Storage Temperature** |
| --- | --- | --- | --- | --- | --- |
| RNALater vs. Plain | GlycerolPBS vs. Plain | 13.333 | 0.00E+00 | 0.00E+00 | 25C |
| GlycerolPBS vs. Plain | Ethanol vs. Plain | -10.824 | 1.56E-18 | 2.12E-16 | 25C |
| Plain vs. Plain | RNALater vs. Plain | -10.562 | 6.67E-16 | 9.07E-14 | 25C |
| GlycerolPBS vs. GlycerolPBS | RNALater vs. Plain | -9.056 | 6.81E-14 | 9.26E-12 | 25C |
| Plain vs. Plain | Ethanol vs. Plain | -8.622 | 1.82E-12 | 2.48E-10 | 25C |
| RNALater vs. Plain | Fresh vs. GlycerolPBS | 8.033 | 9.53E-12 | 1.30E-09 | 25C |
| GlycerolPBS vs. GlycerolPBS | Ethanol vs. Plain | -7.736 | 2.66E-11 | 3.61E-09 | 25C |
| Fresh vs. GlycerolPBS | Ethanol vs. Plain | -6.530 | 6.65E-09 | 9.04E-07 | 25C |
| All within Buffer | RNALater vs. Plain | -6.040 | 9.44E-09 | 1.28E-06 | 25C |
| RNALater vs. Plain | Fresh vs. Plain | 6.145 | 4.33E-08 | 5.89E-06 | 25C |
| RNALater vs. Fresh | RNALater vs. Plain | -5.742 | 1.67E-07 | 2.27E-05 | 25C |
| All within Buffer | Ethanol vs. Plain | -5.433 | 1.90E-07 | 2.58E-05 | 25C |
| RNALater vs. GlycerolPBS | GlycerolPBS vs. Plain | 5.280 | 5.97E-07 | 8.12E-05 | 25C |
| RNALater vs. Plain | Fresh vs. Ethanol | 5.325 | 9.24E-07 | 1.26E-04 | 25C |
| RNALater vs. Fresh | Ethanol vs. Plain | -4.742 | 9.23E-06 | 1.26E-03 | 25C |
| All between Buffer | RNALater vs. Plain | -4.424 | 1.18E-05 | 1.60E-03 | 25C |
| RNALater vs. Ethanol | RNALater vs. Plain | -4.494 | 1.50E-05 | 2.04E-03 | 25C |
| Plain vs. Plain | RNALater vs. GlycerolPBS | -4.548 | 1.79E-05 | 2.43E-03 | 25C |
| Fresh vs. Ethanol | Ethanol vs. Plain | -4.508 | 2.24E-05 | 3.05E-03 | 25C |
| GlycerolPBS vs. GlycerolPBS | RNALater vs. GlycerolPBS | -4.383 | 2.94E-05 | 4.00E-03 | 25C |
| All between Buffer | GlycerolPBS vs. Plain | 4.152 | 3.84E-05 | 5.22E-03 | 25C |
| Fresh vs. Plain | Ethanol vs. Plain | -4.367 | 4.27E-05 | 5.80E-03 | 25C |
| RNALater vs. RNALater | RNALater vs. Plain | -4.295 | 4.48E-05 | 6.09E-03 | 25C |
| All within Buffer | All between Buffer | -4.018 | 6.62E-05 | 9.00E-03 | 25C |
| All between Buffer | Plain vs. Plain | 3.920 | 1.01E-04 | 1.38E-02 | 25C |
| RNALater vs. Ethanol | Ethanol vs. Plain | -3.923 | 1.40E-04 | 1.90E-02 | 25C |
| Fresh vs. Fresh | RNALater vs. Plain | -4.087 | 1.44E-04 | 1.95E-02 | 25C |
| All between Buffer | GlycerolPBS vs. GlycerolPBS | 3.823 | 1.48E-04 | 2.02E-02 | 25C |
| Ethanol vs. Ethanol | RNALater vs. Plain | -3.921 | 1.74E-04 | 2.37E-02 | 25C |
| All between Buffer | Ethanol vs. Plain | -3.752 | 1.94E-04 | 2.65E-02 | 25C |
| All within Buffer | RNALater vs. GlycerolPBS | -3.662 | 3.25E-04 | 4.42E-02 | 25C |
| GlycerolPBS vs. Ethanol | GlycerolPBS vs. Plain | 3.676 | 3.59E-04 | 4.88E-02 | 25C |
| RNALater vs. Plain | GlycerolPBS vs. Ethanol | 8.033 | 9.21E-13 | 1.25E-10 | 4C |
| Ethanol vs. Ethanol | RNALater vs. Plain | -7.455 | 5.92E-11 | 8.05E-09 | 4C |
| RNALater vs. Ethanol | RNALater vs. Plain | -6.880 | 2.13E-10 | 2.89E-08 | 4C |
| GlycerolPBS vs. Ethanol | Ethanol vs. Plain | -6.898 | 3.03E-10 | 4.13E-08 | 4C |
| RNALater vs. Plain | Fresh vs. Ethanol | 7.118 | 4.44E-10 | 6.03E-08 | 4C |
| RNALater vs. Plain | Fresh vs. GlycerolPBS | 7.062 | 7.99E-10 | 1.09E-07 | 4C |
| RNALater vs. GlycerolPBS | RNALater vs. Plain | -6.624 | 1.18E-09 | 1.60E-07 | 4C |
| GlycerolPBS vs. GlycerolPBS | RNALater vs. Plain | -6.885 | 1.70E-09 | 2.31E-07 | 4C |
| All within Buffer | RNALater vs. Plain | -6.355 | 2.00E-09 | 2.73E-07 | 4C |
| Ethanol vs. Ethanol | Ethanol vs. Plain | -6.409 | 7.05E-09 | 9.59E-07 | 4C |
| RNALater vs. Ethanol | Ethanol vs. Plain | -5.926 | 2.52E-08 | 3.42E-06 | 4C |
| All between Buffer | RNALater vs. Plain | -5.459 | 7.54E-08 | 1.03E-05 | 4C |
| RNALater vs. Fresh | RNALater vs. Plain | -5.868 | 9.86E-08 | 1.34E-05 | 4C |
| All within Buffer | Ethanol vs. Plain | -5.502 | 1.43E-07 | 1.94E-05 | 4C |
| GlycerolPBS vs. GlycerolPBS | Ethanol vs. Plain | -5.807 | 1.54E-07 | 2.09E-05 | 4C |
| Fresh vs. Ethanol | Ethanol vs. Plain | -5.684 | 2.12E-07 | 2.88E-05 | 4C |

| **Group 1** | **Group 2** | **t statistic** | **Parametric p-value** | **Parametric p-value (Bonferroni-corrected)** | **Storage Temperature** |
| --- | --- | --- | --- | --- | --- |
| RNALater vs. GlycerolPBS | Ethanol vs. Plain | -5.516 | 2.16E-07 | 2.94E-05 | 4C |
| RNALater vs. RNALater | RNALater vs. Plain | -5.547 | 3.01E-07 | 4.10E-05 | 4C |
| Fresh vs. GlycerolPBS | Ethanol vs. Plain | -5.616 | 3.34E-07 | 4.54E-05 | 4C |
| All between Buffer | Ethanol vs. Plain | -4.480 | 9.26E-06 | 1.26E-03 | 4C |
| RNALater vs. RNALater | Ethanol vs. Plain | -4.674 | 1.06E-05 | 1.44E-03 | 4C |
| RNALater vs. Fresh | Ethanol vs. Plain | -4.565 | 1.81E-05 | 2.46E-03 | 4C |
| GlycerolPBS vs. Ethanol | GlycerolPBS vs. Plain | -4.214 | 5.40E-05 | 7.35E-03 | 4C |
| Ethanol vs. Ethanol | GlycerolPBS vs. Plain | -4.020 | 1.36E-04 | 1.85E-02 | 4C |
| RNALater vs. Plain | GlycerolPBS vs. Ethanol | 9.720 | 0.00E+00 | 0.00E+00 | minus80C |
| GlycerolPBS vs. GlycerolPBS | RNALater vs. Plain | -12.911 | 2.95E-21 | 4.01E-19 | minus80C |
| RNALater vs. RNALater | GlycerolPBS vs. GlycerolPBS | 9.822 | 2.98E-14 | 4.05E-12 | minus80C |
| GlycerolPBS vs. GlycerolPBS | Ethanol vs. Plain | -9.180 | 3.90E-14 | 5.30E-12 | minus80C |
| GlycerolPBS vs. GlycerolPBS | RNALater vs. Fresh | -9.286 | 1.05E-12 | 1.43E-10 | minus80C |
| All between Buffer | GlycerolPBS vs. GlycerolPBS | 7.134 | 3.41E-12 | 4.64E-10 | minus80C |
| GlycerolPBS vs. GlycerolPBS | RNALater vs. GlycerolPBS | -7.593 | 1.85E-11 | 2.52E-09 | minus80C |
| RNALater vs. RNALater | GlycerolPBS vs. Ethanol | 7.489 | 2.17E-11 | 2.95E-09 | minus80C |
| GlycerolPBS vs. GlycerolPBS | RNALater vs. Ethanol | -7.363 | 3.90E-11 | 5.30E-09 | minus80C |
| GlycerolPBS vs. GlycerolPBS | Fresh vs. Plain | -8.661 | 4.63E-11 | 6.30E-09 | minus80C |
| GlycerolPBS vs. GlycerolPBS | Plain vs. Plain | -8.765 | 6.06E-11 | 8.24E-09 | minus80C |
| All between Buffer | GlycerolPBS vs. Ethanol | 6.543 | 1.38E-10 | 1.88E-08 | minus80C |
| GlycerolPBS vs. GlycerolPBS | Fresh vs. GlycerolPBS | -7.933 | 2.11E-10 | 2.87E-08 | minus80C |
| GlycerolPBS vs. Ethanol | Ethanol vs. Plain | -6.862 | 2.88E-10 | 3.91E-08 | minus80C |
| GlycerolPBS vs. GlycerolPBS | Fresh vs. Ethanol | -7.344 | 1.26E-09 | 1.71E-07 | minus80C |
| RNALater vs. Ethanol | GlycerolPBS vs. Ethanol | 6.369 | 2.18E-09 | 2.96E-07 | minus80C |
| RNALater vs. GlycerolPBS | GlycerolPBS vs. Ethanol | 6.342 | 2.84E-09 | 3.86E-07 | minus80C |
| RNALater vs. Plain | Fresh vs. GlycerolPBS | 6.542 | 6.32E-09 | 8.59E-07 | minus80C |
| GlycerolPBS vs. GlycerolPBS | GlycerolPBS vs. Plain | -6.556 | 6.53E-09 | 8.88E-07 | minus80C |
| Ethanol vs. Ethanol | RNALater vs. Plain | -6.138 | 2.35E-08 | 3.19E-06 | minus80C |
| RNALater vs. Fresh | GlycerolPBS vs. Ethanol | 6.055 | 2.66E-08 | 3.62E-06 | minus80C |
| Fresh vs. Fresh | GlycerolPBS vs. GlycerolPBS | 6.821 | 1.72E-07 | 2.34E-05 | minus80C |
| Plain vs. Plain | GlycerolPBS vs. Ethanol | 5.279 | 9.80E-07 | 1.33E-04 | minus80C |
| All within Buffer | RNALater vs. Plain | -4.947 | 1.80E-06 | 2.44E-04 | minus80C |
| All within Buffer | GlycerolPBS vs. GlycerolPBS | 4.687 | 6.35E-06 | 8.64E-04 | minus80C |
| RNALater vs. Plain | Fresh vs. Ethanol | 4.829 | 6.61E-06 | 8.99E-04 | minus80C |
| RNALater vs. RNALater | Fresh vs. GlycerolPBS | 4.886 | 8.49E-06 | 1.15E-03 | minus80C |
| Fresh vs. Plain | GlycerolPBS vs. Ethanol | 4.717 | 8.92E-06 | 1.21E-03 | minus80C |
| GlycerolPBS vs. Ethanol | GlycerolPBS vs. Plain | -4.587 | 1.13E-05 | 1.54E-03 | minus80C |
| RNALater vs. RNALater | Ethanol vs. Ethanol | 4.694 | 1.29E-05 | 1.76E-03 | minus80C |
| Ethanol vs. Ethanol | Ethanol vs. Plain | -4.137 | 8.03E-05 | 1.09E-02 | minus80C |
| All between Buffer | RNALater vs. Plain | -3.899 | 1.09E-04 | 1.48E-02 | minus80C |
| RNALater vs. Fresh | Fresh vs. GlycerolPBS | 4.162 | 1.27E-04 | 1.73E-02 | minus80C |
| Fresh vs. GlycerolPBS | Ethanol vs. Plain | -3.973 | 1.60E-04 | 2.18E-02 | minus80C |
| Plain vs. Plain | Fresh vs. GlycerolPBS | 4.084 | 2.27E-04 | 3.09E-02 | minus80C |
| All between Buffer | Ethanol vs. Ethanol | 3.712 | 2.28E-04 | 3.11E-02 | minus80C |
| Ethanol vs. Ethanol | RNALater vs. Ethanol | -3.797 | 2.36E-04 | 3.21E-02 | minus80C |
| All within Buffer | RNALater vs. RNALater | -3.765 | 2.38E-04 | 3.23E-02 | minus80C |
| Ethanol vs. Ethanol | RNALater vs. GlycerolPBS | -3.777 | 2.63E-04 | 3.57E-02 | minus80C |
